# What determines the switch between atrophic and neovascular forms of age related macular degeneration? - the role of BMP4 induced senescence

**DOI:** 10.18632/aging.100078

**Published:** 2009-08-12

**Authors:** DanHong Zhu, Xuemei Deng, Jing Xu, David R Hinton

**Affiliations:** ^1^ Arnold and Mabel Beckman Macular Research Center, Doheny Eye Institute, Los Angeles, CA 90033, USA; ^2^ Departments of Pathology, Keck School of Medicine of the University of Southern California, Los Angeles CA 90089, USA; ^3^ Departments of Ophthalmology, Keck School of Medicine of the University of Southern California, Los Angeles CA 90089, USA

**Keywords:** BMP4, age related macular degeneration, senescence, retinal pigment epithelial cell, oxidative stress

## Abstract

Age-related macular degeneration (AMD), the leading cause of blindness in the elderly, targets the retinal pigment epithelium (RPE), a monolayer of cells at the back of the eye. As AMD progresses, it can develop into two distinct forms of late AMD: "dry," atrophic AMD, characterized by RPE senescence and geographic RPE loss, and "wet," neovascular AMD, characterized by RPE activation with abnormal growth of choroidal vessels. The genetic and molecular pathways that lead to these diverse phenotypes are currently under investigation. We have found that bone morphogenetic protein-4 (BMP4) is differentially expressed in atrophic and neovascular AMD. In atrophic AMD, BMP4 is highly expressed in RPE, and mediates oxidative stress induced RPE senescencein vitro via Smad and p38 pathways. In contrast, in neovascular AMD lesions, BMP4 expression in RPE is low, possibly a result of local expression of pro-inflammatory mediators. Thus, BMP4 may be involved in the molecular switch determining which phenotypic pathway is taken in the progression of AMD.

Age-related macular degeneration (AMD) is the leading cause of irreversible blindness in the elderly [1-2]. Considerable evidence supports the opinion that the retinal pigment epithelium (RPE), a monolayer of cells between the light sensitive photoreceptors and the vascular choroid, is a primary site of pathology in the disease [1-5]. The RPE provides support for the photoreceptors and plays a critical role in the visual cycle; thus, degeneration and loss of RPE lead to secondary degeneration of photoreceptor cells [3]. Early AMD is characterized by the presence of extracellular deposits, or drusen, beneath the RPE. Increasing numbers of large drusen predispose to the development of the late blinding forms of the disease which can manifest in two disparate ways. In late, "dry" AMD, geographic loss of RPE occurs in the macular region, while in the late neovascular or "wet" form of the disease, there is abnormal growth of choroidal vessels under the retina which leak fluid and may progress to form a disciform scar (Figure 1) [1-5]. Pathogenic mechanisms for AMD include both genetic and environmentalfactors related to primary RPE senescence, alterations in the complement pathway, increased inflammation, changes in the balance of growth factors, excessive lipofuscinaccumulation, and oxidative stress [5]. Major genetic risk factors for AMD, including Complement Factor H and HTRA1 variants, appear to predispose to both atrophic and neovascular AMD [6,7]; only recently has a genetic variant been identified that specifically predisposes to the atrophic form [8]. Consequently, there is considerable interest in further establishing the factors that mediate the "molecular switch" that may determine which late form of the disease an individual develops.

Recently, we reported that bone morphogenetic protein (BMP)4 is prominently expressed in the RPE and adjacent extracellular matrix of patients with the dry or atrophic form of AMD when compared to controls (Figure 2A, B). Here, we show that in the wet or neovascular form of the disease (5 patients with surgical excision of choroidal neovascular membranes due to neovascular AMD) there is almost no expression of BMP4 in the RPE and adjacent neovascular tissues (Figure 2C). Interestingly, in cases (3 patients) in which the neovascular lesion had progressed to a fibrous scar, the level of BMP4 expression increased in the RPE and adjacent tissues (Figure 2D). This has led us to the hypothesis that BMP4 may be a molecular switch participating in the pathway decision that determines which form of late AMD develops.

**Figure 1.**
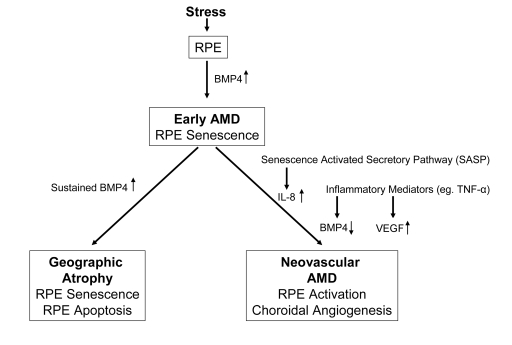
Diagram illustrating the progression of early age related macular degeneration (AMD) into 2 divergent late stages and the potential role of BMP4 as a switch between these pathways. Chronic stressors such as oxidative stress can promote the expression of BMP4 in the retinal pigment epithelium (RPE) and induce RPE senescence as part of the phenotype of early AMD. If BMP4 expression is sustained, it could lead to RPE apoptosis and geographic atrophy. In other individuals, activation of the senescence activated secretory pathway and expression of pro-inflammatory mediators could result in increased expression of interleukin (IL)-8, decreased expression of BMP4 and increased expression of vascular endothelial growth factor (VEGF) resulting in neovascular AMD with choroidal angiogenesis.

BMP4 is an important regulator of differentiation, senescence and apoptosis in many different cells and tissues [9,10]. We reported that BMP4 can induce RPE senescence *in vitro* [11], and that RPE chronically exposed to sublethal doses of oxidative stress can increase their BMP4 expression and exhibit a senescent phenotype, thus supporting the contention that BMP4 mediates oxidative stress-induced RPE senescence. We further determined that BMP4 mediates RPE senescence via activation of Smad and p38 pathways to activate p53, and increase expression of p21^WAF1/ cip1^, and to decrease phospho-Rb. Importantly, BMP4-mediated RPE senescence can be inhibited by Chordin-like, a BMP4 antagonist, and SB203580, a phospho-p38 inhibitor. Our findings not only disclose a molecular pathway linking oxidative stress with RPE senescence, but also provide a novel therapeutic target for treatment of atrophic AMD.

**Figure 2.**
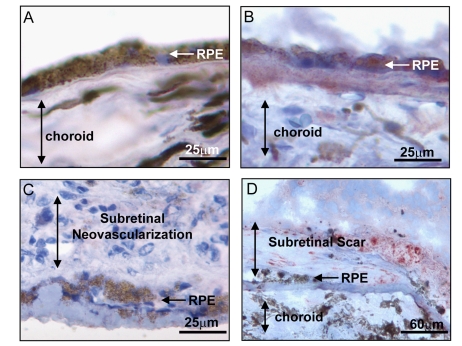
Expression of BMP4 in late stages of age related macular degeneration (AMD). Immunohistochemical stains for BMP4 (red chromogen) in retinal pigment epithelium (RPE)/choroid tissue sections from donor eyes with hematoxylin counterstain. In (**A**) a control individual without AMD shows no apparent BMP4 staining in RPE or choroid. In (**B**) an individual with late dry AMD, away from a region of geographic atrophy shows prominent BMP4 immunoreactivity in RPE and in the accumulated drusen material between the RPE and the choroid. In (**C**) an individual with neovascular form of late AMD shows no apparent BMP4 staining in the RPE or the neovascular lesion between the RPE and retina. In (**D**) an individual with neovascular form of late AMD that further progressed to scar with loss of neovascular channels shows re-expression of BMP4 staining in cells within and adjacent to the lesion. Note loss of most cells in RPE layer. The institutional review board (IRB) of the University of Southern California approved our use of human donor eyes. All procedures conformed to the Declaration of Helsinki forresearch involving human subjects.

Recently, Demidenko et al. [12] evaluated the concept that duration of cell cycle arrest determines the progressive loss of proliferative capacity characteristic of cellular senescence [12]. Using a variety of cell lines including the spontaneously immortalized human RPE cell line, ARPE-19, they found that rapamycin, an inhibitor of the nutrient-sensor mammalian target of rapamycin (mTOR), partially prevented loss of proliferative potential induced by oxidative stress, or ectopic p21 or p16 exposure, leading to deceleration of cellular senescence [12]. This work supports the critical role of oxidative stress, and cell cycle arrest in induction of senescence and demonstrates a pharmacologic approach to suppression of RPE senescence [12].

Interestingly, BMP4 has been found to be involved in chemotherapeutic agent-induced premature senescence of cancer cells [13]. Adriamycin and BMP4 treatment can induce lung cancer cell senescence, and BMP4 expression is increased in Adriamycin-treated lung cancer cells. This BMP4-induced premature senescence is mediated through Smad signaling to up-regulate p16^INK4a^ and p21^WAF1/ cip1^. BMP4 and other BMP signaling pathways were also found to participate in senescence of multiple cancer cell types or in the inhibition of tumor cell growth [14,15]. For example, BMP-2 and -4 inhibit prostate cancer cell growth through Smad-1 phosphorylation, p21^WAF1/ cip1^ up-regulation, and Rb dephosphorylation, while in glioblastoma, BMP4 and its cognate receptors can trigger the Smad signaling cascade to reduce the proliferation of tumor cells [16]. Together, these studies reveal that BMP4 induces and mediates the premature senescence of both malignant cells in tumors and aging RPE cells in dry AMD.

**Figure 3.**
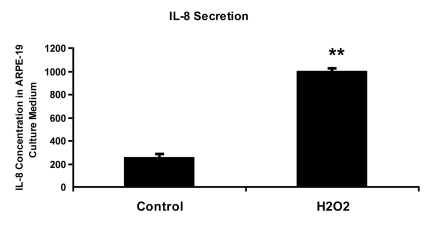
IL-8 protein concentration in culture medium measured by ELISA. ARPE-19 cells were treated with 150 uM H_2_O_2_ in culture medium with 10% fetal bovine serum for 2 hours and allowed to recover in stressor-free ARPE medium for 22 hours. The procedure was repeated to generate the next treatment cycle. The twice treated cells were allowed to stay in 1% serum ARPE medium for 72 hours after stress before proceeding to further analytic assays. The culture media from control and senescent RPE cells were collected and used directly for ELISA measurement. IL-8 secretion level was measured in pg/ml using human IL-8 ELISA Kit (BioLegend, Inc., San Diego, CA) according to manufacturer's instructions. The level of IL-8 secretion shown here was averaged from a triplicate of each sample and from 3 independent repeats of H_2_O_2_ treatments. Student's t test was used for statistical analysis (**; p < 0.0005).

Transforming growth factor (TGF)-β has been extensively reported to be involved in mediating oxidative stress induced premature senescence of fibroblasts [17-19]. Recently it has been reported that TGF-β mediates oxidative stress induced RPE cell senescence through the up-regulation of p21^ WAF1/ cip1^ and the down-regulation of phosphorylated Rb and that blockade of TGF-β signaling by specific TGF-β antibody can impede RPE senescence [20]. This finding is similar to our finding for BMP4 mediated oxidative stress-induced RPE senescence. We suggest that TGF-β and BMP4 may have a synergistic effect in mediating the oxidative stress-induced RPE senescence, because neither TGF-β antibodies nor BMP4 antagonist alone can completely block the expression of senescence marker genes to baseline in the oxidative stress treated RPE cells. More investigations are needed to elucidate the interactions between TGF-β and BMP signaling cascades in oxidative stress-induced RPE senescence.

A variety of intrinsic and extrinsic stress signals can activate the p53 pathway, which then triggers either cellular senescence or apoptosis [21,22]. We found that both BMP4 and oxidant treatment can increase p53 protein level in RPE cells. A microarray analysis of the RPE transcriptome from the maculas of six healthy, elderly human donors revealed a statistically significant overrepresentation of genes associated with stress, with the p53 gene listed in the top 30 most highly expressed RPE genes [23]. Although little is known about how p53 regulates cellular senescence and how p53 interacts with the BMP-Smad pathway, the fact that p53 levels were increased in RPE cells after BMP4 treatment and Smad1/5 could bind to p53 [24], raises the possibility that Smad1/5 activates p53 dependent transcription through the regulation of post-translational modifications of p53, such as phosphorylation and acetylation.

It remains unanswered why some patients develop atrophic AMD while others develop the neovascular form of the disease. The switch between dry and wet AMD may be related to differences in the microenvironment created by senescent RPE cells, which secrete a number of cytokines and growth factors [25]. The defined components of this "senescence associated secretory phenotype" (SASP) include elements associated with inflammation, and angiogenesis, such as interleukin (IL)-6 and IL-8 [26-28]. We have found that RPE cells induced into senescence by chronic oxidative stress secrete 4 times higher IL-8 than non-senescent RPE cells (Figure 3). IL-8 promotes angiogenesis by increasing the proliferation, survival and migration of endothelial cells and promotes inflammation by increasing neutrophil chemotaxis and degranulation [29-31]. Together these findings suggest that chronic oxidative stress increases the premature senescence of RPE. If RPE do not go down the cell death pathway to atrophic AMD, the senescent RPE may secrete high levels of IL-8, which in turn stimulate inflammation and angiogenesis. But what about the finding that neovascular AMD lesions show minimal levels of BMP4? In other cell types, pro-inflammatory mediators such as tumor necrosis factor (TNF)-alpha have been shown to downregulate BMP4 expression [32]. In the absence of BMP4, neovascular endothelial cells, stimulated by increased expression of vascular endothelial growth factor, and without the growth inhibitory senescence and cell death effects mediated by BMP4, would be in a permissive environ-ment for angiogenesis [5]. This idea is further supported by the finding that when neovascular AMD lesions undergo subsequent scar formation, with degeneration and loss of neovascular endothelial cells, there is a concomitant increase in BMP4 expression (Figure 2).

It has been previously observed that tissues in aged individuals may exhibit the paradoxical juxtaposition of atrophy and hyperplasia within the same tissue or even within the same cell type [33]. This response may be explained in part by senescent heterogeneity [34,35]. *In vitro* culture of human fibroblasts results in a fraction of cells senescing at every population doubling. The senescent cells have shorter telomeres than their cycling counterparts. Thus, it was concluded that the main cause of intrinsic heterogeneity of senescent fibroblasts was the cell to cell variation of telomere shortening [36]. Using pulse-chase 5-bromodeoxyuridine-labeling assay, Gonzalez and colleagues revealed that the senescent heart contained functionally competent cardiac progenitor cells (CPCs) with longer telomeres, and these stem cell-like CPCs can be activated and migrate to the damaged regions to generate a population of young cardiomyocytes and partly reverse the aging myopathy [37].

Much remains to be learned about the genetic and environmental factors mediating the progression of early AMD to its late forms. Our finding of differential expression of BMP4 in geographic atrophy and neovascular AMD and the interactive roles of oxidative stress, inflammation and senescence in the regulation and functional effects of this growth factor, suggests the possibility that BMP4 may be playing a part in the molecular switch determining which phenotypic pathway is taken in the progression of AMD.

## Acknowledgments

This work was supported by National Institutes of Health grants EY01545 and EY03040 and by the Arnold and Mabel Beckman Foundation.
